# Visceral Inflammation and Immune Activation Stress the Brain

**DOI:** 10.3389/fimmu.2017.01613

**Published:** 2017-11-22

**Authors:** Peter Holzer, Aitak Farzi, Ahmed M. Hassan, Geraldine Zenz, Angela Jačan, Florian Reichmann

**Affiliations:** ^1^Research Unit of Translational Neurogastroenterology, Institute of Experimental and Clinical Pharmacology, Medical University of Graz, Graz, Austria; ^2^BioTechMed-Graz, Graz, Austria; ^3^CBmed GmbH—Center for Biomarker Research in Medicine, Graz, Austria

**Keywords:** gut–brain axis, gut microbiota, immune–brain axis, immune stress, intestinal inflammation, lipopolysaccharide, mental health, neuropeptide Y

## Abstract

Stress refers to a dynamic process in which the homeostasis of an organism is challenged, the outcome depending on the type, severity, and duration of stressors involved, the stress responses triggered, and the stress resilience of the organism. Importantly, the relationship between stress and the immune system is bidirectional, as not only stressors have an impact on immune function, but alterations in immune function themselves can elicit stress responses. Such bidirectional interactions have been prominently identified to occur in the gastrointestinal tract in which there is a close cross-talk between the gut microbiota and the local immune system, governed by the permeability of the intestinal mucosa. External stressors disturb the homeostasis between microbiota and gut, these disturbances being signaled to the brain *via* multiple communication pathways constituting the gut–brain axis, ultimately eliciting stress responses and perturbations of brain function. In view of these relationships, the present article sets out to highlight some of the interactions between peripheral immune activation, especially in the visceral system, and brain function, behavior, and stress coping. These issues are exemplified by the way through which the intestinal microbiota as well as microbe-associated molecular patterns including lipopolysaccharide communicate with the immune system and brain, and the mechanisms whereby overt inflammation in the GI tract impacts on emotional-affective behavior, pain sensitivity, and stress coping. The interactions between the peripheral immune system and the brain take place along the gut–brain axis, the major communication pathways of which comprise microbial metabolites, gut hormones, immune mediators, and sensory neurons. Through these signaling systems, several transmitter and neuropeptide systems within the brain are altered under conditions of peripheral immune stress, enabling adaptive processes related to stress coping and resilience to take place. These aspects of the impact of immune stress on molecular and behavioral processes in the brain have a bearing on several disturbances of mental health and highlight novel opportunities of therapeutic intervention.

## Introduction

In a general context, stress is considered to be a dynamic process in which the physical and/or mental homeostasis of an organism is challenged, the outcome depending on the type, severity, and duration of stimuli (stressors) involved, the stress responses triggered and the stress susceptibility/resilience of the organism. Homeostatic disturbances can be triggered by both exogenous and endogenous stressors. There is abundant evidence that the immune system is involved in stress responses, given that both physical and psychosocial stressors have an impact on immune function. It needs to be emphasized, however, that the interaction between stress and immune system is a bidirectional process, implying that alterations in immune function themselves can elicit stress responses. Such bidirectional interactions have been identified to occur in the gastrointestinal (GI) tract in which there is a close cross-talk between the gut microbiota and the local immune system (1–4), governed by the permeability of the GI mucosa. On the one hand, external stressors impact on the gut microbiota and its relationship with the GI mucosal, immune, endocrine, and nervous system. On the other hand, this disturbance of gut homeostasis is signaled to the central nervous system (CNS) *via* multiple communication pathways constituting the gut–brain axis, ultimately eliciting stress responses and perturbations of brain function (5).

It has been known for some time that infection-related as well as infection-independent immunological stimuli can evoke stress responses as reflected by an increased activity of the hypothalamic–pituitary–adrenal (HPA) axis, resulting in enhanced plasma concentrations of adrenocorticotropic hormone (ACTH) and cortisol/corticosterone (6, 7). Pathogen-associated molecular patterns (PAMPs) such as bacterial lipopolysaccharide (LPS) have been extensively studied in their ability to stimulate the innate immune system *via* binding to toll-like receptor-4 (TLR4), cause the formation of proinflammatory cytokines, activate the HPA system (8–10), and alter brain function and behavior. Cytokines generated in response to, e.g., LPS trigger a complex behavioral response, encompassed in the terms “sickness behavior” or “illness response,” which comprise fever, anorexia, somnolence, decrease in locomotion, exploration and social interaction, hyperalgesia, and delayed depression-like behavior (11–15). These cerebral effects are brought about by multiple signaling mechanisms: direct access of cytokines to the brain, activation of vagal afferent neurons, and neuroinflammatory processes in the brain (11, 12, 14, 16). Once acute sickness subsides, depression-like behavior may ensue, in which cytokine-induced HPA axis hyperactivity plays a particular role (17).

Given the abundance of the gut microbiota (18), it is commonly assumed that a large part of the circulating levels of LPS and related PAMPs derive from bacteria in the GI tract (19) and that the effects of intraperitoneally (IP) administered LPS replicate primarily the reactions to increased translocation of LPS from the gut lumen under conditions of enhanced mucosal permeability. The intestinal mucosal barrier is subject to many influences that regulate its cellular and paracellular permeability, among which stress is an important factor. de Punder and Pruimboom (19) hypothesize that the stress-induced increase in mucosal permeability serves to meet the enhanced metabolic demand under conditions of stress. At the same time, a persistent increase in the translocation of LPS to the circulation is associated with pathologies such as chronic GI inflammation (20) and non-alcoholic fatty liver disease (21) but also with chronic fatigue syndrome (22), depression (23), and autism spectrum disorder (24). A minor part of circulating PAMPs may also derive from other microbe-colonized organs, such as oral cavity, respiratory system, and genitourinary tract as well as from food (19). It has, in addition, been argued that there are dormant bacterial reservoirs in the blood and certain tissues, including the brain, and that PAMP production in these reservoirs may contribute to chronic inflammatory disease (25).

In view of these facts and conditions, the present article sets out to highlight some of the interactions between peripheral immune activation, especially in the visceral system, and brain function, behavior, and stress coping. These issues are exemplified by the way the intestinal microbiota and its metabolites communicate with the immune system and CNS, on the one hand, and the mechanisms whereby overt inflammation in the GI tract impacts on brain function, pain sensitivity, and stress coping, on the other hand. As the interactions between the peripheral immune system and the brain take place along the gut–brain axis, the major pathways of this communication system are also briefly dealt with. Furthermore, novel insights into the molecular signaling processes in the brain that occur under conditions of peripheral immune stress are discussed, and adaptive processes related to stress coping and resilience are considered. In concluding, these novel aspects of immune–brain interaction are put into perspective with disturbances of mental health that become manifest under conditions of stress and with emerging opportunities of therapeutic intervention.

## Multiple Communication Pathways Along the Gut–Brain Axis

**Figure 1:**
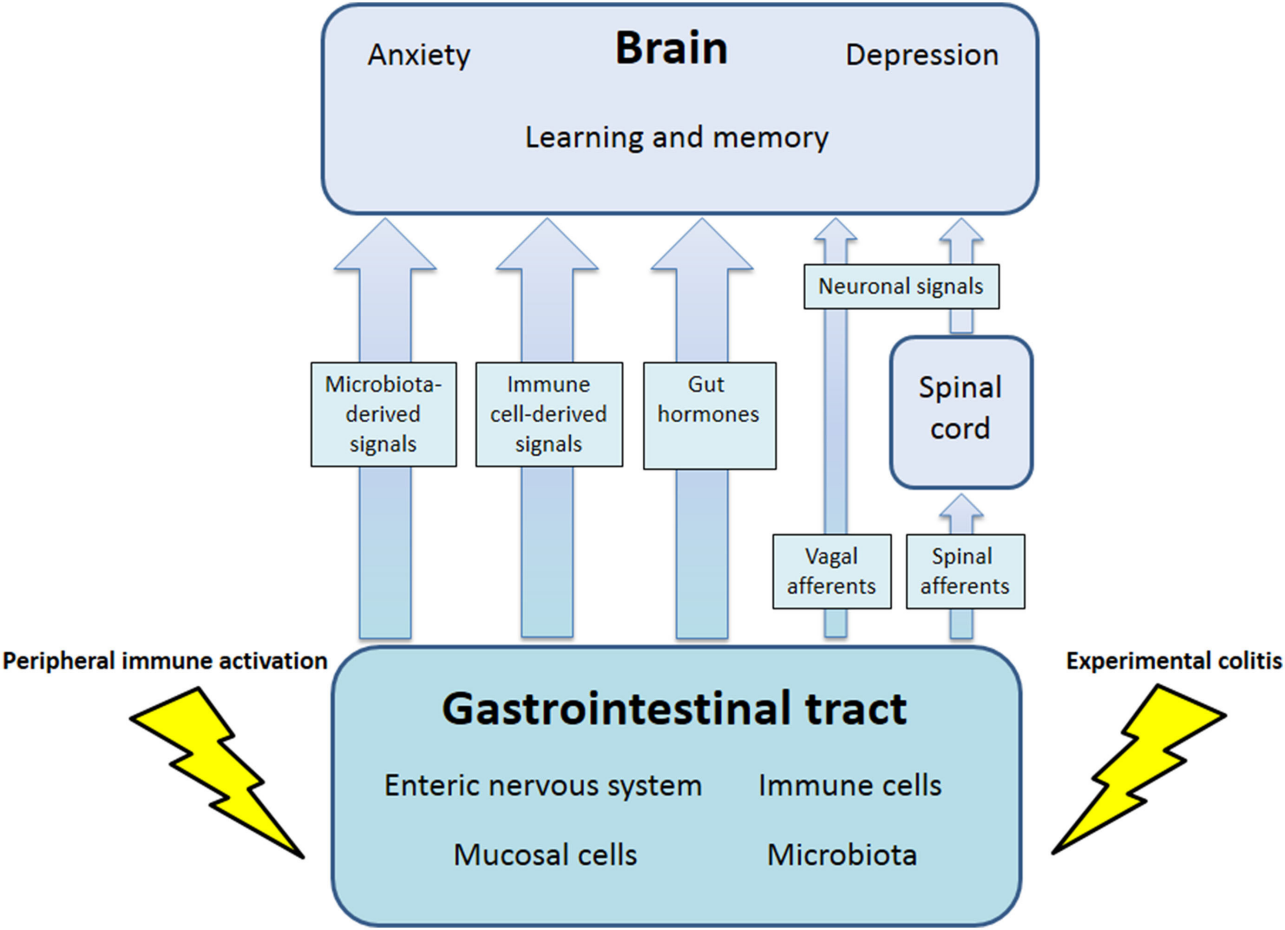
Pathways involved in the behavioral disturbances associated with visceral immune activation and inflammation. There are multiple communication pathways between gut and brain: microbiota-derived signals, immune cell-derived signals, gut hormones, and vagal and spinal afferents. In the course of experimental colitis or microbe-evoked peripheral immune activation, signaling along these pathways is altered, ultimately influencing brain functions, such as anxiety, depression-like behavior, learning, and memory.

The communication network between GI microbiota, mucosa, endocrine system, immune system, and enteric nervous system, on the one hand, and the brain, on the other hand, uses at least five information carriers (Figure 1): gut microbiota-derived molecules, immune mediators, gut hormones, vagal afferent neurons, and spinal afferent neurons (5). As the interaction between gut and the brain is bidirectional, there are also at least four information carriers that signal from the CNS to the GI tract: parasympathetic efferent neurons, sympathetic efferent neurons, neuroendocrine factors involving the adrenal medulla, and neuroendocrine factors involving the adrenal cortex (5). Additional relays include the blood–brain barrier (BBB) and distinct brain circuits that process the information the CNS receives from the periphery.

It is important to note that these circulation-based (endocrine) and neuronal communication routes do not operate in isolation but are closely interrelated with each other. Microbial metabolites, microbe-associated molecular patterns (MAMPs), and PAMPs can act both on GI endocrine and/or immune cells and sensory neurons. This is exemplified by the short-chain fatty acids (SCFAs) comprising acetic, *n*-butyric, and propionic acid, which are generated from otherwise indigestible carbohydrate fibers through microbial fermentation. SCFAs are multi-target messengers that act on GI endocrine, mucosal, and immune cells as well as on brain microglia (26, 27). SCFAs are important energy sources for the microbiota and mucosa, exert antiinflammatory effects through their action on macrophages, neutrophils, dendritic, and regulatory T cells, and have a fortifying influence on the GI epithelial barrier (27) as well as BBB (28).

Most of the cellular effects of SCFAs are mediated by G protein-coupled receptors (GPRs), such as GPR41 (also known as FFAR3), GPR43 (also known as FFAR2), and GPR109A (also known as HCAR2) (26, 27). This is also true for the impact SCFAs have on enteroendocrine cells in the GI mucosa. By stimulating GPR41 and GPR43 on L cells in the distal ileum and colon, SCFAs release the gut hormones peptide YY (PYY), glucagon-like peptide-1 (GLP-1), and GLP-2 (29–31). Through this route, enteroendocrine cells convey messages of the gut microbiota within the digestive system as well as to distant organs, including the brain. Following their release from L cells, PYY and GLP-1 inhibit gastric motility, improve glucose homeostasis, induce satiety (29, 32), and alter behavior (33, 34). It is likely that other appetite-regulating hormones, such as ghrelin, cholecystokinin, and leptin, are also under the influence of the gut microbiota (35–37). Gut hormone activity may be coupled with intestinal immune processes, as proinflammatory prostaglandins (PGs) acting *via* EP_4_ receptors enhance the release of GLP-1, GLP-2, and PYY (38), and enteroendocrine cell activity is increased in Crohn’s disease affecting the small bowel (39). In addition, enteroendocrine cells may be involved in hormone-independent ways of gut–brain communication, given that misfolded α-synuclein could be transferred to the brain through direct connections between enteroendocrine cells and neural circuits, thus contributing to the pathogenesis of Parkinson’s disease (40).

A number of gut hormones including PYY, GLP-1, and ghrelin signal to the brain to affect appetite and energy homeostasis but also impact on mood and emotional-affective behavior (29, 32–34). This messaging is not only accomplished *via* a circulatory route but also through activation of vagal afferent neurons (5, 41). The vagus nerve appears in fact to play a particular role in the signaling of microbial, endocrine, and immune signals to the brain, which is consistent with its predominant sensory nature, given that the vast majority (80–90%) of the axons in the vagus nerve are afferent nerve fibers (42–44). Vagal afferents are thought to tonically deliver information from visceral organs to the brain, this massive sensory input being relevant not only to the autonomic regulation of digestion and energy balance but also to interoception (45, 46). Also termed the “sixth sense” (45), interoception refers to the integrated sense of the physiological condition of the body (46) and the representation of the internal state in the brain (47). GI interoception includes a wide range of conscious sensations, such as pain, nausea, GI discomfort, GI tension, hunger, and thirst, as well as signaling processes that go virtually unnoticed although they impact on emotional-affective and cognitive processes (48–50).

## Immune Stress Signaling from the Gut to the Brain

### Immune Signaling *via* Microbial Factors

The gut microbiota is a rich source of potential messenger molecules: primary metabolites generated by microbial cells, MAMPs, and PAMPs shed from microbial cells, and secondary metabolites generated by microbial fermentation of food components or transformation of host molecules such as bile acids (27, 51–55). Apart from the MAMPs and PAMPs, many of the other microbial metabolites, such as SCFAs, trimethylamine-*N*-oxide, p-cresol, aryl hydrocarbon receptor ligands, formyl peptides, flagellin, polyamines such as spermidine, 4-ethyl phenol sulfate (4-EPS), and polysaccharide A produced by *Bacteroides fragilis* (27), have an effect on the immune system, may influence sensory nerve activity or travel by the blood stream to distant organs including the brain (Table 1). While we know many factors that govern the composition, diversity, and function of the gut microbiota, we still lack a full comprehension of the signaling systems that govern the homeostatic interaction between the gut microbiota and the local immune system as well as the resilience of this homeostatic cross-talk. In a wider perspective, a dysbalance in the micobiota–immune relationship represents itself a stress scenario which, if this “immune stress” is transmitted to the brain, will elicit a systemic stress response. As alluded to before, the intestinal mucosal barrier (27) plays an important role in the interaction between the gut microbiota and the intestinal immune system. Spadoni and colleagues (56) have recently characterized some of the structural and functional characteristics of the gut–vascular barrier in mice and humans that controls the translocation of microbial macromolecules into the bloodstream and denies entry of microbial cells. They identified Wnt/β-catenin signaling in gut endothelial cells as an important control mechanism which, when downregulated, may enable certain pathogenic bacteria such as *Salmonella typhimurium* to enter the bloodstream (56).

**Table 1:** Effects of PAMPs and other microbial metabolites on emotional-affective and cognitive behavior.

PAMP/metabolite	Main receptor	Dose	Species (sex)	Behavioral effects	Additional effects	Reference
MALP-2 FSL-1	TLR2/6	100 µg/kg IP	Wistar rats (male)	Sickness behavior: anorexia, adipsia, hypoactivity	Hypo- and hyperthermia, upregulated levels of proinflammatory cytokines in plasma	(137)

Pam3CSK4	TLR2/1	200 ng/2 μl in mice, 1 μg/3 μl in rats, ICV	Mice and rats (male)	Sickness behavior: anorexia, hypoactivity	Hypothalamic inflammation and microglia activation, increased POMC neuron activity, hyperthermia	(138)

LTA	TLR2	20 mg/kg IP	C57BL/6N mice (male)	No effect 3 h after LTA injection	Upregulated levels of proinflammatory cytokines in plasma (proteins) and brain (mRNA), decreased expression of tight junction-associated proteins in the brain, increased circulating corticosterone levels	(97)

LPS	TLR4	0.83 mg/kg IP	C57BL/6 mice (male)	Acute sickness (6 h) and delayed depression (24 h)	Expression of acute (c-Fos) and chronic (ΔFosB) cellular reactivity markers	(13)
			Crl:CD1 mice (male)	Depression-like behavior prevented by minocycline or IDO antagonist 1-MT	Enhanced kynurenine/tryptophan ratio in plasma and brain normalized by minocycline or 1-MT	(191)
		0.8 ng/kg IV	Healthy human volunteers (male)	Anxiety, depressed mood, and decreased memory performance	Increased circulating levels of IL-6, TNF-α, soluble TNF receptors, IL-1 receptor antagonist and cortisol, mild increase in rectal temperature	(87)
		0.4 ng/kg IV	Healthy human volunteers (male, female)	Anxiety, depressed mood, sickness symptoms	Increase in circulating proinflammatory cytokines and cortisol higher in females than males	(90)
		1.0 ng/kg IV	Healthy human volunteers (male)	Sickness symptoms	Microglial activation throughout the brain, increased circulating levels of proinflammatory cytokines	(91)

FK565, MDP, LPS	NOD1, NOD2, TLR4	3, 3, 0.1 mg/kg IP	C57BL/6 mice (male)	NOD agonists alone without effect, synergism with LPS in eliciting sickness	Hypothermia, upregulated levels of proinflammatory cytokines in plasma (proteins) and brain (mRNA), increased circulating corticosterone levels	(10)

Poly I:C	TLR3	6 mg/kg IP	Sprague–Dawley rats (male)	Reduced locomotor activity (6 h), anxiety-like behavior (24 h), reduced saccharin preference (24–72 h)	Decreased body weight gain (24 h), molecular changes in frontal cortex and hippocampus: increased proinflammatory cytokine and IDO expression (mRNA, 6 h), reduced BDNF and TrkB expression (mRNA, 6, 24, 48 h), increased tryptophan (6, 24, 48 h), and kynurenine (24, 48 h) levels	(174)
		2, 6, 12 mg/kg IP	C57BL/6 mice (female)	Dose-dependent acute sickness observed in OFT (4, 8, 12 h) and burrowing (6, 10, 26 h)	Upregulation of proinflammatory cytokines in plasma (protein) and brain (mRNA), biphasic core body temperature change	(96)
		12 mg/kg IP	C57BL/6J mice (male)	Deficit in contextual memory consolidation (24 h)	Diminished BDNF mRNA expression (4 h)	(176)

4-EPS		30 mg/kg IP for 3 weeks	C57BL/6N mice	Increased anxiety and startle reflex	Increase in 4-EPS levels in response to maternal immune activation by Poly I:C	(57)
SCFAs	GPR41	25 mM sodium propionate, 40 mM sodium butyrate plus 67.5 mM sodium acetate in drinking water for 7 weeks	BDF1 mice overexpressing α-synuclein	Motor deficits	α-Synuclein-mediated neuroinflammation	(82)
	GPR43	25 mM sodium propionate, 40 mM sodium butyrate plus 67.5 mM sodium acetate in drinking water for 4 weeks	Germ-free C57BL/6 mice (male and female)		Normalization of microglia density, morphology and immaturity (altered in germ-free mice)	(26)

Sodium butyrate	GPR41	1 g/kg by oral gavage for 3 days	Germ-free C57BL/6J mice (male)	Normalization of blood–brain barrier permeability which is enhanced in germ-free mice	Normalization of occludin expression in frontal cortex which is decreased in germ-free mice, increase of histone acetylation in brain lysates	(28)
	GPR43	1.2 g/kg IP in single injection or for 4 weeks	C57BL/6J mice	Antidepressant-like effect	Increase of histone acetylation in hippocampus	(83)
		1.2 g/kg IP	Aged (24 months) Wistar rats (male)	Rescue of aging-associated memory impairment		(84)

Propionic acid	GPR41	4 µl of 0.26 M solution, ICV	Adolescent (41 days) Long–Evans rats (male)	Restricted behavioral interest in a specific object, impaired social behavior, impaired reversal in T-maze task	Neuroinflammatory response	(85)
	GPR43	4 µl of 0.26 M solution ICV for 8 days	Long-Evans rats	Increase of locomotor activity	Change in molecular phospholipid species in blood and brain	(86)

*BDNF, brain-derived neurotrophic factor; 4-EPS, 4-ethyl phenol sulfate; FSL-1; fibroblast-stimulating lipopeptide-1; ICV, intracerebroventricular; IDO, indoleamine 2,3-dioxygenase; IL, interleukin; IP, intraperitoneal; IV, intravenous; LTA, lipoteichoic acid; LPS, lipopolysaccharide; MALP-2, macrophage-activating lipopeptide-2; MDP, muramyl dipeptide; 1-MT, 1-methyltryptophan; NOD, nucleotide-binding and oligomerization domain; OFT, open field test; PAMP, pathogen-associated molecular pattern; poly I:C, polyinosinic:polycytidylic acid; POMC, proopiomelanocortin; SCFA, short-chain fatty acid; TLR, Toll-like receptor; TNF, tumor necrosis factor; TrkB, tropomyosin-related kinase B*.

Although the list of identified chemical messengers derived from the gut microbiota is steadily increasing, only a limited number of these molecules have been investigated in their effects on gut–brain and immune–brain signaling: PAMPs such as LPS, lipoteichoic acid (LTA), and peptidoglycan components, SCFAs and 4-EPS (Table 1). The latter metabolite is markedly increased in a mouse model of autism spectrum disorder which is caused by maternal immune activation and characterized by enhanced gut permeability, altered microbial composition, altered serum metabolomic profile, and defects in communicative, stereotypic, anxiety-like, and sensorimotor behaviors (57). Some of these behavioral abnormalities are reproduced by 4-EPS, while treatment with the human commensal *Bacteroides fragilis* has a beneficial effect (57).

### Immune Signaling Across the Blood-Brain Barrier (BBB)

The BBB is an important checkpoint for the entry of molecules and cells into the brain and in this capacity shares many similarities with the gut–vascular barrier (56, 58). Both boundaries are built by a cellular layer that controls the movement of molecules and cells and closely interacts with neighboring immune and other cells that provide functional support to the barrier (56, 58). In the present context, it is particularly worth noting that SCFAs play not only a role in the gut epithelial barrier but also in the development and maintenance of the BBB. This implication has been disclosed in germ-free mice in which increased BBB permeability is associated with reduced expression of the tight junction proteins occludin and claudin-5 in frontal cortex, striatum, and hippocampus (28). A decrease in the expression of these tight junction proteins in the murine hippocampus, but not amygdala, prefrontal cortex, and hypothalamus, has likewise been found after antibiotic-induced disruption of the gut microbiota (54). Re-colonization of the intestine of germ-free adult mice with a normal gut microbiota normalizes BBB permeability and upregulates the expression of tight junction proteins, an effect that is reproduced by butyric acid (28). The microbial control of BBB development and function has very likely a bearing on gut–brain and particularly immune–brain signaling, because the transfer of immune-relevant factors (e.g., cytokines, chemokines, PGs) and even immune cells across the BBB depends on the functional status of the barrier and its regulatory mechanisms (14, 59). Given that the BBB is essential for brain development, function, and homeostasis, the control of BBB permeability is probably an important mechanism whereby the gut microbiota controls brain activity and behavior.

### Immune Signaling *via* the Vagus Nerve

As alluded to before, immune signaling from the gut to the brain can also take a neuronal route, particularly *via* the vagus nerve. Microbial as well as immune factors appear to alter the excitability and activity of both enteric sensory as well as vagal afferent neurons, which appear to be connected with each other *via* junctions involving nicotinic acetylcholine receptors (60, 61). One such factor is polysaccharide A derived from *Bacteroides fragilis* which stimulates sensory neurons of the myenteric plexus (62) while components of *Lactobacillus rhamnosus* (JB-1) have a similar stimulant effect on vagal afferent neurons (63). Such microbe-driven neuronal processes are likely to participate in the vagus nerve-dependent effects of probiotics on brain function and behavior (64, 65).

The role of the abdominal vagus in transmitting microbial and immune messages to the brainstem is related both to the proximity of vagal afferent nerve fibers to immunologically relevant structures in the abdominal cavity (11, 16) and to the sensitivity of these nerve fibers to messengers derived from the microbiota and immune system. It has been shown, for instance, that IP administered LPS is primarily transported to the liver where it induces the release of interleukin (IL)-1β from Kupffer cells (macrophage-like cells to screen blood and lymph) (11, 16). The cytokine, in turn, is thought to excite afferent nerve fibers in the hepatic branch of the vagus nerve or to enhance their afferent signaling (11, 66). In addition, the abdominal vagus is associated with paraganglia and connective tissue containing macrophages and dendritic cells that respond to IP administration of LPS with synthesis of IL-1β (67, 68). The abdominal paraganglia of the vagus nerve contain glomus-like cells that have IL-1 receptors (69), are innervated by vagal afferents (70), and appear to act as chemosensory accessory cells (16). Furthermore, vagal afferents innervate abdominal lymph nodes that represent another interface with the visceral immune system (16). Unlike endocrine signaling *via* the bloodstream, sensory neurons are, thus, in a position to enable rapid propagation of immune signals to the brain.

The sensitivity of vagal afferent nerve fibers to PAMPs such as LPS and proinflammatory cytokines has been corroborated by electrophysiological recordings and c-Fos mapping in the central projection areas of sensory neurons. In addition, it has been shown that both vagal and spinal afferent neurons do not only respond to these microbial and immune messengers but can also, under their influence, undergo sensitization to other stimulants. For instance, both LPS and tumor necrosis factor-α (TNF- α) are capable of directly activating vagal afferent neurons in culture (71). In addition, LPS can stimulate sensory neurons *via* activation of the transient receptor potential ankyrin-1 (TRPA1) ion channel (72) and sensitize afferent fibers in mesenteric nerves to serotonin, bradykinin, and gut distension, an effect in which mast cells and cyclooxygenase-2 play a role (73). In a similar manner, IL-1β is able to increase action potential firing in vagal afferents (74, 75), to induce c-Fos expression in the nucleus tractus solitarii, the central projection area of vagal afferent nerve fibers in the medullary brainstem (76), and to sensitize vagal afferent pathways to gastric acid (77). The expression of IL-1 receptors by nodose ganglion cells makes it likely that the cytokine is capable of exciting vagal afferents by a direct action on the axons, although PGs acting *via* EP_3_ receptors and cholecystokinin acting *via* CCK_1_ receptors have also been implicated (75, 78). Spinal afferent neurons supplying the murine colon are also responsive to proinflammatory cytokines, such as IL-1β and TNF-α, and the mechanical hypersensitivity of mouse colonic nerve fibers evoked by TNF-α is inhibited by a TRPA1 blocker (79).

## Impact of Immune Stress Signaling from the Periphery on Brain Function and Behavior

As discussed before, microbial and immune messages originating from the visceral system reach the brain either by an endocrine or neuronal route, the BBB being an important checkpoint for those messengers that arrive *via* the bloodstream. Sensitization of CNS pathways as well as long-term alterations in brain circuitry, connectivity, and activity are ultimately responsible for the mental disturbances in which immune activation and chronic inflammatory disease appear to play a role. The impact of MAMPs and PAMPs, particularly LPS, on the brain *via* particular immune pathways has been most extensively studied in this respect, although a contribution of other factors, such as 4-EPS (57), spermidine (80), and SCFAs is also emerging (Table 1). Apart from disturbances of brain function and behavior, PAMPs acting *via* the pattern recognition receptor (PRR) TLR4 (such as LPS) also seem to contribute to the pathogenesis of cerebrovascular disease (81). Specifically, Gram-negative bacteria of the gut microbiota and TLR4 activation stimulate the formation of cerebral cavernous malformations (CCMs) that are risk factors for stroke and seizure. Activation of TLR4 by LPS accelerates CCM formation in mice, which in turn is prevented by genetic or pharmacological blockade of TLR4 signaling (81).

Short chain fatty acids can enter the brain through uptake by monocarboxylate transporters at the BBB (53). In the brain, SCFAs support the maturation and function of microglial cells which are the resident macrophages of the CNS (26). In a transgenic mouse model of Parkinson’s disease, however, SCFAs trigger a microglia-dependent immune response, enhance α-synuclein aggregation, and elicit movement disturbances (82). Injected systemically to mice, butyrate induces an antidepressant-like behavioral response which is associated with an increased expression of brain-derived neurotrophic factor (BDNF) (83). Butyrate is also able to ameliorate the memory decline that develops in aging rats (84), while administration of propionate to rodents has been shown to evoke behavioral abnormalities reminiscent of autism spectrum disorder (85, 86). These findings indicate that microbiota-derived signaling molecules can have both beneficial and deleterious effects on brain function and behavior, the outcome depending very likely on both microbe and host factors.

While most information on the cerebral impact of PAMP/MAMP-evoked immune stimulation has been derived from animal studies, select microbial metabolites, such as LPS, have also been tested in humans. For instance, intravenous LPS injection in healthy human volunteers increases the circulating levels of IL-6, IL-10, TNF-α, soluble TNF receptor, IL-1 receptor antagonist, and cortisol, which is associated with enhanced body temperature, anxiety, negative mood, decreased memory performance, and hyperalgesia (87–90). While these effects are similar to those observed in rodents, the potency of LPS in terms of dose per body weight is >100 times higher in humans (88). Mechanistic studies have shown that the sickness response elicited by intravenous LPS injection in healthy male humans is associated with microglial activation throughout the brain as observed by positron emission tomography (91).

Table 1 summarizes a number of studies in which the effects of PAMPs, MAMPs, and some other microbial metabolites on behavior and related molecular changes have been investigated in rodents and humans. In judging the relevance of these effects it is important to take account of the doses studied and the species, strain, and sex of the subjects tested. Males and females differ in both innate and adaptive immune responses (92) and these sex differences also extend to PAMP/MAMP reactions. For instance, macrophages of male mice express higher levels of TLR4 on their cell surface than those of females, which may explain why male mice respond to LPS with formation of more IL-6 than females (93). The additive effect of LPS and muramyl dipeptide (MDP) to attenuate locomotion is likewise more pronounced in female than male rats (94). Similar observations have been made in humans, given that women react to LPS with enhanced release of proinflammatory cytokines, cortisol, and prolactin compared to males (90). Despite these sex differences, men and women do not differ in LPS-evoked anxiety, mood depression, and sickness, which points to compensatory mechanisms that balance the cerebral impact of the exaggerated immune response in women (90). Sex differences may also influence the pharmacokinetics and pharmacodynamics of immune responses to microbial metabolites (95), and the molecular targets and mechanisms of action of PAMPs/MAMPs may considerably differ with dose (10, 14, 96, 97). This is true for LPS that at the lower dose range induces various dimensions of the sickness reponse as well as depression-like behavior (10, 14) whereas, at a higher dose range, it causes septic shock.

### Cytokines As Mediators of LPS-Induced Effects on the Brain

Immune stress signaling across the BBB evokes a neuroinflammatory reaction in the CNS, which contributes to the behavioral disturbances associated with peripheral immune activation. The underlying processes have been most extensively studied with LPS, a PAMP known to target a variety of immune and other cells *via* stimulation of TLR4. At doses <1 mg/kg, LPS reproducibly evokes acute sickness which may evolve into depression-like behavior about 24 h after its injection (13, 98, 99). Chronic exposure to LPS for 8 weeks exerts similar behavioral effects (100). There is some evidence that LPS may induce neuroinflammation also by a mechanism involving NOD-like receptor pyrin domain-containing protein-3 (NLRP3) inflammasome activation and in this way cause long-term deficits in affective and cognitive behavior (101). Although the NLRP3 inflammasome inhibitor Ac-Tyr-Val-Ala-Asp-chloromethylketone prevents the LPS-induced effects on brain and behavior (101), the high dose of LPS used (5 mg/kg) may have caused sepsis-like effects that limit the interpretation of the findings.

Through activation of TLR4, peripheral administration of LPS leads to increased expression of proinflammatory cytokines in the periphery and brain (10, 102, 103). Among these proinflammatory cytokines, IL-1β and TNF-α are considered to be the predominant mediators of LPS-induced sickness behavior, while other cytokines, such as IL-6 and IFN-γ, are thought to primarily amplify the effects of IL-1β and TNF-α (14, 104). Circulating TNFα and IL-1β interact with their receptors on cerebral endothelial cells (CECs), induce the production of cytokines and other secondary messengers, such as PG and nitric oxide (NO), and thereby modulate CNS function and behavior (105–107). Proinflammatory cytokines can also access the brain *via* structures that lack a BBB, such as the circumventricular organs and the choroid plexus, and thus alter brain function (108). Furthermore, monocyte chemoattractants such as monocyte chemoattractant protein-1 (MCP-1/CCL2), which can be expressed by circumventricular organs or activated microglia in response to proinflammatory cytokines, can promote monocyte migration into the brain (103).

Peripheral administration of LPS, IL-1β, or TNF-α induces cytokine expression within the brain and leads to the full spectrum of sickness behavior (109, 110). However, when both IL-1β and TNF-α are blocked, LPS-induced sickness is attenuated (104, 111). IL-1β and TNF-α have been demonstrated to exert their behavioral effects *via* activation of the IL-1 receptor of type I (111) and TNF receptor of type 1 (112), respectively. Both receptors are expressed on neurons and other cell types of the CNS and have been proposed to impact on behavior ultimately through direct effects on neuronal activity (104). However, type-1 TNF receptor signaling on astrocytes has been shown to modify hippocampal excitatory synapses and impair contextual learning/memory through an astrocyte–neuron signaling cascade involving presynaptic *N*-methyl-d-aspartate (NMDA)-type glutamate receptors containing the NR2B subunit (113). On the neuronal level, prolonged exposure to TNF-α is able to inhibit long-term potentiation in hippocampal slices (114). In hippocampal neuronal cultures, TNF-α is able to evoke neuronal excitation through activation of sphingomyelin phosphodiesterase 3 and production of ceramide, an intracellular signaling molecule that leads to NMDA receptor-mediated calcium influx (115). This process enhances the insertion of NMDA receptors containing NR1 subunits into the cell membrane and increases the rate and amplitude of NMDA-receptor-mediated calcium bursts. In addition, the mitogen-activated protein kinase (MAPK) signaling pathway is stimulated by TNF-α, given that inhibition of c-jun N-terminal kinase blocks TNF-α-induced sickness (116). The MAPK pathway may also be responsible for the effect of TNF-α to induce depression-like behavior at intracerebroventricular (ICV) doses that are too low to induce signs of sickness (117, 118).

The transduction mechanisms operated by IL-1β in the CNS comprise the MAPK pathway (119, 120) and inhibition of neuronal long-term potentiation by inhibiting calcium channels (121, 122) but also induction of neuronal hyperexcitation *via* formation of ceramide (123). Moreover, activation of the IL-1 receptor stimulates the mTOR pathway and leads to synaptic defects through increased levels of the epigenetic regulator methyl-CpG binding protein 2 (124). Some of the adverse effects of IL-1β in the brain may also result from its ability to impair long-distance signaling of BDNF by attenuating retrograde endosome trafficking (125). Unlike IL-1β, acute ICV injection of IL-6 does not induce sickness behavior, although it is able to induce fever and activate the HPA axis (126). In addition, IL-6 is required for the manifestation of a full sickness response, and genetic deletion of IL-6 blunts the sickness response to LPS and IL-1β (127). The differences in the behavioral effects of IL-1β and IL-6 have been attributed to the apparent inability of IL-6 to stimulate ceramide synthesis (104) although IL-6 is able to activate similar signaling pathways as IL-1β and TNF-α, leading to a reinforcement of proinflammatory cytokine formation. There is also information that the induction of IL-6 by LPS may differ from that of other cytokines. Thus, while LPS causes an early stimulation of nuclear factor κB (NF-κB), activation of the transduction factor NF-IL-6, which contributes to the expression of IL-6 (128), reaches its peak only 8 h post-treatment (129). An involvement of NF-IL-6 in the delayed inflammatory and behavioral response to LPS has been confirmed by genetic deletion of NF-IL-6, the response being reversed 24 h after LPS treatment (130).

When used as a treatment for cancer or hepatitis C, IFN-α induces signs of sickness (fatigue, decreased motivation, reduced appetite, altered sleep) in nearly all patients within the first week, later followed by the development of symptoms of major depression (sadness, decreased mood, anhedonia, impaired cognitive function) in 30–50% of the patients (131). Analysis of potential vulnerability factors has shown that the patients at risk to develop major depression experience a threefold higher increase of circulating ACTH and cortisol levels in response to the first administration of IFN-α than resilient patients (17). Peripheral (132) and central (133) administration of IFN-α to mice causes depression-like behavior (133), and long-term administration of LPS to rats produces a specific cytokine response in the brain characterized by increased IL-1β and IFN-γ levels (100). Inoculation of mice with Bacille Calmette–Guérin, an attenuated form of *Mycobacterium bovis*, induces depression-like behavior, an effect that is absent in IFN-γ receptor knockout mice while an acute episode of sickness behavior persists (134).

### Behavioral Effects of Bacterial PAMPs and MAMPs Other Than LPS

In its effect on the brain, LPS is joined by many bacterial, viral, and fungal PAMPs and MAMPs (Table 1). Peptidoglycan, for instance, is a cell wall constituent of many bacteria that has been demonstrated to stimulate the innate immune system and modulate behavior. While peptidoglycan is a TLR2 agonist, its fragments γ-d-glutamyl-meso-diaminopimelic acid (iE-DAP) and MDP activate the intracellular nucleotide-binding and oligomerization domain (NOD) innate immune receptors NOD1 and NOD2, respectively (135). Furthermore, the family of antibacterial pattern recognition molecules termed peptidoglycan recognition proteins (PGRPs) typically binds the muramyl pentapeptide, or tetrapeptide fragment of peptidoglycan (136). In a mechanistic perspective, it is relevant to know that TLR2 can heterodimerize with TLR1 and TLR6, forming TLR2/1 and TLR2/6 heterodimers, respectively. IP injection of the TLR2/6 agonists macrophage-activating lipopeptide-2 or the synthetic analog fibroblast-stimulating lipopeptide-1 induces cytokine release and sickness behavior in rats (137). Similarly, ICV injection of the TLR2/1 agonist Pam3CSK4 evokes sickness which can be attenuated by NF-κB or COX inhibition with indomethacin (138). In addition, TLR2/1 activation causes hypothalamic inflammation and microglia activation, increases physical contacts between microglia and proopiomelanocortin (POMC) neurons, and stimulates the activity of POMC neurons (138). In line with these hypothalamic effects, Pam3CSK4-induced anorexia can be reversed by ICV administration of a melanocortin receptor 3/4 antagonist (138).

Exposure to peptidoglycan early in life, either during pregnancy or postnatally, has adverse effects on the brain. Intravenous injection of peptidoglycan into pregnant dams traverses the placenta and reaches the fetal brain where it causes marked neuronal proliferation through TLR2/6 agonism (139). This fetal neuroproliferative response which involves the neuronal nuclear transcription factor FoxG1 is associated with abnormal cognitive behavior in the pups following birth (139). Exposure of the postnatal mouse brain to the TLR2/1 agonist Pam3CSK4 or TLR2/6 agonist fibroblast-stimulating lipopeptide-1 has a life-long effect on learning and memory, spatial memory being impaired by the TLR2/1 agonist only, while contextual and cued fear learning in adult mice is enhanced by both agonists (140). Peptidoglycan derived from the gut microbiota can also translocate into the brain and activate cerebral PRRs (141). Specifically, the levels of peptidoglycan in the developing brain parallel the postnatal microbial colonization of the gut, while peptidoglycan-sensing molecules (TLR2, NODs, PGRPs) are expressed during early postnatal brain development and altered in response to perturbation of the gut microbiota (141). Knockout of the PGRP 2 is associated with enhanced sociability and alterations in the expression of the autism risk gene *c-Met*, the synaptogenesis marker *Synaptophysin* and *Bdnf* (141). Germ-free mice are likewise more sociable as reported by the same laboratory (142), whereas Desbonnet et al. (143) find decreased sociability in germ-free mice. Besides acting on the brain, peptidoglycan derived from the gut microbiota also impacts on the systemic immune system, given that it stimulates and primes bone marrow-derived neutrophils through NOD1 (144).

Lipoteichoic acid is a cell wall component of Gram-positive bacteria, which activates primarily TLR2 and in this way causes peripheral immune activation and initiates neuroinflammatory processes in the mouse brain that are associated with downregulation of BBB components and activation of the HPA axis, although emotional behavior is not affected (97). Many commercial LTA preparations are potentially contaminated with LPS, in which case the biological effects of LTA preparations can in part be attributed to the presence of LPS or a particular interaction between TLR2 and TLR4 activation (97). Such positive or negative interactions between different PAMPs in their effects on immune system and brain are of pathophysiological relevance because bacteria are usually equipped with a multitude of PAMPs. NOD agonists, for instance, evoke only mild immune stimulation on their own but synergize with LPS and lead to aggravated cytokine expression and sickness behavior (10). In the colon, however, the interaction of NOD and TLR4 is antagonistic, with NOD2 activation attenuating TLR4-dependent cytokine production and experimental colitis (145).

While activation of PRRs induces behavioral disturbances, inactivation of these receptors can also cause behavioral deficits. Thus, TLR4 knockout mice exhibit reduced novelty-seeking and social interaction in an approach-avoidance conflict situation, a deficit that can be reversed by administration of GABA into the nucleus accumbens shell which appears to be hyperactivated in response to behavioral testing of TLR4 knockout mice (146). TLR2 knockout mice display reduced exploratory behavior, impaired spatial learning, and enhanced contextual and cued fear learning (140). While Park et al. (147) likewise report cognitive impairment in TLR2 knockout mice, they also observe schizophrenia-like symptoms, such as hyperlocomotion, anxiolytic-like behavior, prepulse inhibition deficits, and social withdrawal. These behavioral perturbations and the associated biochemical alterations (increased p-Akt and p-GSK-3α/β expression) are reversed by antipsychotic drug administration (147). Double deletion of the *Tlr2* and *Tlr4* genes is associated with decreased exploratory behavior and impaired performance in a visual discrimination reversal task (148).

### Behavioral Effects of Viral PAMPs

While research on the impact of immune stress on the brain has thus far been focused on bacterial immune stimulants, interest in the contribution of viral immune stimulants is also increasing. It is well recognized that psychological stress can adversely influence the outbreak and recurrence of Herpes simplex virus, human immunodeficiency virus (HIV), and hepatitis C virus (HCV) infections (149, 150) *via* activation of the HPA axis and sympathetic adrenomedullary system (151, 152). Vice versa, HIV seropositive patients may suffer from many neuropsychiatric disorders, in particular major depression and dementia (153). Like HCV (154), HIV can cross the BBB simply by infecting macrophages that migrate to the brain where the virus leads to neurotoxin-mediated neuronal loss and synaptic damage (155, 156).

The PAMPs presented by viruses are DNA and RNA molecules as well as surface glycoproteins (157, 158). Nucleic acids of viral origin are recognized by TLR3, TLR7, TLR8, and TLR9 in endosomal compartments, as well as by RNA helicase retinoic acid-inducible gene-I (RIG-I) and melanoma-differentiation-associated gene 5 (MDA5) found in the cytosol. Receptor stimulation leads to a proinflammatory immune response by activating a battery of transcription factors followed by the production of type I IFNs and other cytokines including IL-1β, IL-6, TNF-α, and IFN-γ, depending on the kind of viral infection (157, 159–161). Furthermore, sensing of viral nucleic acids by RIG-I and other PRRs leads to inflammasome formation and activation of caspase-1, resulting in the production of IL-1β and IL-18, enhancement of the antiviral response of the immune system and pyroptosis (162, 163). The cytokines formed in response to viral infection signal to the brain, evoke sickness behavior and cause other perturbations of brain function and behavior. The effect of the influenza virus to depress food intake in mice, for instance, is attenuated by an IL-1 receptor antagonist (164). In humans, the plasma levels of IL-1β are elevated in patients suffering from post-viral depression when compared to patients who do not develop post-viral depression (165).

Polyinosinic:polycytidylic acid (Poly I:C) is a synthetic PAMP that is widely used to study virus-evoked stimulation of the innate immune system, as it specifically mimics dsRNA molecules occurring during the replication of most viruses except negative-strand RNA viruses (166). Like dsRNA, Poly I:C binds to TLR3 in endosomal compartments (160, 167) and, to a much lesser extent, to cytosolic RIG-I as well as MDA5 if the molecule contains more than 2,000 base pairs (168). Systemic Poly I:C administration to rodents induces expression and release of proinflammatory cytokines, especially type I IFNs, IL-6, IL-1β, and TNF-α (96, 160), the magnitude of effect being considerably smaller than that evoked by the TLR4 agonist LPS (169). Since Poly I:C is rapidly degraded by RNases (170), its prolonged neuroinflammatory and behavioral effects are mediated by the cytokines it induces (171, 172).

Poly I:C given to adult rodents mimics the acute phase of a viral infection, alters a variety of brain functions and gives rise to a sequence of behavioral alterations (Table 1). Within 4 h, Poly I:C causes fever and attenuates activity, followed by anxiety-like behavior and anhedonic depression-like behavior that can be observed during the period 24–72 h post-treatment (96, 173, 174). In addition, an impairment of working memory and a deficit in contextual fear memory consolidation become manifest (175, 176). These behavioral perturbations are associated with disruption of the BBB, enhanced expression of indoleamine 2,3-dioxygenase (IDO) in hippocampus and frontal cortex, suppression of hippocampal neurogenesis and decreased expression of BDNF in hippocampus and cortex (173, 174, 176, 177). In line with the common notion that systemic infection can impair cognitive function, administration of Poly I:C over several days increases amyloid-β_1–42_ levels in hippocampal tissues, paralleled by a deficit in hippocampus-dependent learning tasks (178). Further analysis has revealed that peripheral immune activation by Poly I:C causes dendritic spine loss and impairs dendritic spine formation associated with learning (179). The resulting deficit in multiple learning tasks in mice is mediated by cells derived from peripheral monocytes and a TNF-α-dependent mechanism but does not require microglial function in the CNS (179). Furthermore, systemic Poly I:C amplifies brain pathology in the ME7 model of prion disease and accelerates progression of neurodegeneration (180). In confirmation of the hypothesis that a viral infection during pregnancy is a risk factor for particular neuropsychiatric diseases in the offspring (181), Poly I:C administration to pregnant rodents has been found to evoke a schizophrenia-like phenotype (182–184) as well as depression-like phenotype (185) in the adult offspring, changes that are associated with hippocampal synaptic deficits in the absence of microglial alterations (186).

### Signaling Molecules in the Brain Affected by Peripheral Immune Stress

IFN-γ is a strong inducer of the tryptophan-degrading enzyme IDO (187) which has gained center stage as a factor in cytokine-induced mood disorders. Cytokine-induced activation of IDO causes augmented conversion of tryptophan to kynurenine. While kynurenine itself is inactive, its metabolites, 3-hydroxykynurenine and quinolinic acid exert neurotoxic effects through NMDA receptor agonism and generation of oxidative radicals (188). By contrast, kynurenic acid, another kynurenine metabolite, is neuroprotective, acting as an antagonist of NMDA and α7 nicotinic acetylcholine receptors (188). Depressed patients display in fact a reduction of kynurenic acid/3-hydroxykynurenine and/or kynurenic acid/quinolinic acid ratios (189). Kynurenine levels rise not only in response to proinflammatory cytokines but also in response to corticosteroids, given that multiple mRNA transcripts of IDO are differentially expressed in response to different immune stimulants and corticosteroids (190). Importantly, the IDO antagonist 1-methyl-d,l-tryptophan is able to block LPS-induced depression-like behavior, while the levels of proinflammatory cytokines and sickness behavior remain unchanged (191). In a similar vein, the NMDA receptor antagonist ketamine abrogates LPS-induced depression-like behavior without affecting the sickness response (192). Ketamine is able to induce rapid antidepressant effects in patients with treatment-refractory depression, highlighting the potential of targeting glutamatergic neurotransmission as a treatment option for depressive disorders (193).

The sickness behavior evoked by immune stimulation comprises anorexia, a response in which IL-18 plays a role through inhibition of type III GABAergic neurons in the bed nucleus of the stria terminalis (194). Secretion of the “satiety hormone” leptin in response to LPS also contributes to inflammation-induced anorexia. Thus, leptin deficiency attenuates LPS-induced anorexia, and further analysis has shown that both the PI3K and STAT3 signaling pathways in leptin receptor-expressing cells are required for the acute hypophagic response to LPS (195). In addition, leptin is involved in cytokine-induced depression, as ICV leptin administration induces depression-like behavior, while leptin antagonism attenuates particular components of this behavioral response (196). Apart from cytokines and leptin, PGs also mediate some aspects of the sickness in response to immune activation. PGE_2_, for instance, has been implicated in inflammation-induced feelings of malaise and discomfort (197). An analogous behavioral readout in mice, LPS-induced conditioned place aversion, is mediated through MyD88-dependent activation of CECs, resulting in cyclooxygenase-1 (COX-1)-dependent PGE_2_ synthesis. By activating EP_1_ receptors on striatal neurons, PGE_2_ inhibits a motivational circuitry through GABA-mediated inhibition of dopaminergic neurons (197).

Cytokines induce a wide range of neurochemical changes in the brain including altered function of monoamine, glutamate, and neuropeptide systems as well as deficits in growth factors such as BDNF (198). Neuropeptide Y (NPY) signaling *via* Y2 and Y4 receptors has in particular been implicated in the short-and long-term behavioral effects of peripheral immune challenge. Thus, Y2 receptor knockout mice are particularly sensitive to the effects of LPS-evoked immune stress to attenuate locomotion and social interaction and to increase anxiety-like behavior, while the LPS-induced rise of circulating corticosterone is suppressed by Y2 receptor knockout (8, 98, 99). Furthermore, knockout of Y2 and Y4 receptors unmasks the ability of a single LPS injection to cause a delayed and prolonged increase in depression-like behavior, which indicates that NPY signaling conveys resilience to some of the adverse effects of immune stress on the brain (98). In addition, combined deletion of NPY and PYY aggravates and prolongs the weight loss caused by Bacille Calmette–Guérin, which attests to an important role of NPY and PYY in orchestrating homeostatic reactions to infection and immune stimulation (199). Changes in serotoninergic and glutamatergic systems seem to be prominently involved in cytokine-induced mood and cognitive symptoms, while the dopamine system has been implicated in symptoms such as fatigue, decreased motivation and altered appetite (200). Cytokine-induced activation of the enzyme GTP cyclohydrolase I (GTP-CH1) is an important mechanism whereby immune activation affects dopamine and serotonin synthesis. Thus, GTP-CH1 activation inhibits the formation of tetrahydrobiopterin which is an essential co-factor of dopamine and serotonin biosynthesis (201). In addition, RNA-Seq studies reveal wide ranging changes of the microglial transcriptome associated with depression-like behavior recorded 7 days after peripheral immune activation by Bacille Calmette–Guérin (202).

Emerging evidence indicates that bioactive lipids, including eicosanoids, endocannabinoids, and specialized pro-resolving mediators (resolvins, maresins, protectins, lipoxins) participate in the regulation of the neuroinflammatory, cerebral and behavioral consequences of immune activation and inflammation (203). PGs are very likely involved in the development of sickness and depression-like behavior induced by peripheral immune challenge. In particular, PGE_2_ is synthesized in the brain in response to a variety of immune signals such as LPS or cytokines, and its administration has been found to evoke sickness behavior through stimulation of EP_2_ receptors (204). Conditional deletion of EP_2_ receptors in myeloid linage cells blunts the brain microglial response to systemic LPS injection, which attests to a role of EP_2_ receptor stimulation in immune–brain signaling (205). Chemoattractant receptor-homologous molecule expressed on T helper type 2 cells (CRTH2) is a PG D_2_ receptor that is also involved in the behavioral effects of LPS (206, 207). Inhibition of PG synthesis by nonsteroidal antiinflammatory drugs attenuates the sickness and depression-like behavior induced by LPS, Bacille Calmette–Guérin and interferon-α-2b (204, 208–211). Cyclooxygenase-2 inhibitors, particularly celecoxib, have the potential to ameliorate depression in humans, although a meta-analysis of the pertinent clinical trials points out that the subgroup of patients who could benefit from such therapy has not yet been identified (212).

Endocannabinoids such as 2-arachidonoylglycerol and anandamide constitute another class of lipid mediators that have an impact at several levels of the immune–brain axis (213). Apart from their inhibitory effect on inflammation in the GI tract (5, 213, 214), endocannabinoids influence the responsiveness of vagal and spinal afferent neurons and modify neuronal as well as microglial activity in the CNS. Activation of cannabinoid CB1 receptors which are expressed by visceral afferent neurons in the vagus nerve (215) blocks the effect of TNF to amplify vagal afferent responsiveness (216). In the brain, endocannabinoids appear to protect from BBB dysfunction and neuroinflammatory processes under conditions of immune challenge. Such a homeostatic role is, for instance, deduced from the finding that hydrolysis of the endocannabinoid 2-arachidonoylglycerol by monoacylglycerol lipase generates neuroinflammatory PGs in response to peripheral LPS administration (217, 218). Prevention of the hydrolysis of the endocannabinoid anandamide by an inhibitor of fatty acid amide hydrolyase likewise attenuates the expression of pro- and antiinflammatory cytokines in the frontal cortex and hippocampus of rats following peripheral stimulation of TLR3 (poly I:C) or TLR4 (LPS) and inhibits the LPS-induced anhedonia, but not sickness response (219, 220). Moreover, deletion of CB2 receptors exacerbates, while CB2 receptor agonism attenuates, stress-induced neuroinflammatory responses in the brain (221). These observations are in line with the emerging concept that the endocannbinoid system in the brain operates at the intersection between stress, immune activation, neuroinflammation, emotionality, and neuropsychiatric disease (222, 223).

Specialized pro-resolving mediators, such as resolvins, maresins, protectins, and lipoxins, play not only a role in the resolution of inflammation but also emerge as regulators of neuroinflammatory processes and their impact on brain function and behavior (203, 224). The antiinflammatory effect of resolvins appears to involve neurons, given that the ability of resolvin D1 (RvD1) to attenuate zymosan-evoked peritonitis in mice depends on the vagus nerve, since vagotomy increases the severity of inflammation (224). This implication of the vagus nerve appears to be mediated by netrin-1, an axonal guidance molecule (224). Both RvD1 and resolvin E1 (RvE1) are able to decrease the LPS-evoked expression of proinflammatory cytokines (TNF-α, IL-6, and IL-1β) in microglia cells *in vitro* (225). The underlying mechanisms differ, however, as RvE1 regulates the NFκB signaling pathway and RvD1 acts via microRNA expression (225). *In vivo* experiments demonstrate that RvD1 and RvD2 counteract the depressogenic effect of LPS *via* the mammalian target of rapamycin complex 1 signaling pathway, an effect in which the medial prefrontal cortex and dentate gyrus are of particular relevance (226). A similar antidepressant effect of RvD1 and RvD2 has been observed in the chronic unpredictable stress model (227). The ability of other specialized pro-resolving mediators such as lipoxins, protectins and maresins to regulate neuroinflammatory processes and behavioral reactions to immune stimulation has not yet been systematically investigated. Systemic administration of lipoxin A4 to mice reduces anxiety-related behavior (228), and treatment of rats with an analog of lipoxin A4 has been reported to attenuate neuroinflammation in a rat model of ischemic stroke and to promote sensorimotor recovery (229). Docosahexaenoic acid is a major n-3 polyunsaturated fatty acid present in the brain, and acute ICV infusion of unesterified docosahexaenoic acid protects from LPS-evoked neuroinflammation, an action that involves conversion to specialized pro-resolving mediators such as neuroprotectin D1 (230).

## Cytokine Hypothesis of Depression

The changes in emotional-affective behavior seen in experimental studies of immune stress and in therapeutic trials of interferon therapy are consistent with the cytokine hypothesis of depression. Following its first formulation as macrophage theory of depression by Smith (231), proinflammatory cytokines as well as microglial activation and neuroinflammation have been demonstrated to occur not only in depression but also in other psychiatric disorders such as schizophrenia and bipolar disorder (232–235). In identifying the precise Research Domain Criteria driven by inflammation it has been recognized that inflammatory processes represent pivotal factors in the psychopathology of symptom dimensions shared by different psychiatric conditions (200). In testing the therapeutic potential of antiinflammatory agents in depressed patients (236), the TNF-α blocker infliximab has been found to improve symptoms only in patients with high inflammatory markers, while patients with low levels of peripheral inflammation experience rather negative effects (237). A similar response pattern has been reported for the treatment with omega-3 fatty acids (238). Low levels of TLR3 in peripheral blood mononuclear cells of depressed patients also predict therapeutic efficacy of antidepressant drugs while high expression of this PRR is associated with treatment failure (239). These clinical observations are in part supported by experimental findings that low cytokine concentrations have a positive influence on learning, memory, and synaptic plasticity (240), that meninges-derived T-cells exert beneficial effects on learning behavior through IL-4 and BDNF expression (241), and that adaptive immunity of the meninges supports cerebral circuits underlying social behavior through IFN-γ (242).

## Implications of the Gut Microbiota in Mood Disorders

The cytokine hypothesis of depression has been extended by an increasing number of observations to suggest that the gut microbiota contributes to the pathogenesis of depression, given that depressed patients may present with a change in the microbial community structure, a disruption of the intestinal barrier and increased endotoxinemia (243). Depression-associated changes in the gut microbial community have been observed at the phylum level, with either a decrease (244, 245) or increase (246) in the relative abundance of *Bacteroidetes*. A decrease in *Bacteroidetes* has also been observed in other disorders of the gut–brain axis such as obesity and irritable bowel syndrome (IBS) (247, 248), although comorbid depression in IBS patients is not associated with particular alterations in the microbial profile (249). A causal relationship between gut microbiota and depression has been inferred from the observations that transplantation of fecal microbiota from depressed patients to germ-free or germ-depleted rodents induces a depression-like phenotype in the animals (245, 250). This transfer of a depression-like pathology is accompanied by an increase in the kynurenine/tryptophan ratio and other inflammation markers (250) and an altered profile of metabolites involved in carbohydrate and amino acid metabolism (245).

Given the impact of stress on mood disorders, stress-induced alterations in the commensal microbiota community, GI barrier and GI physiology are increasingly recognized to be relevant to stress-evoked immune activation and behavioral disturbances (251). It has long been known that stress enhances the permeability of the GI mucosa (19), and there is now increasing evidence that a dysfunctional intestinal barrier enables microbiota-driven immune activation to impact on the brain (252). Stress-evoked alterations of the microbial community toward a composition favoring immune stimulation may exacerbate the adverse influence of gut leakiness on the immune–brain interaction. Stress exposure not only disrupts the community structure of the gut microbiota (253) but also induces translocation of, for instance, *Lactobacillus* spp. to the spleen and primes the innate immune system for enhanced reactivity (254).

Antibiotic-induced depletion of the intestinal microbiota blocks stress-induced bacterial translocation and, consequently, TLR activation and neuroinflammation in the brain (255). Antibiotic treatment as well as neutralization of LPS also blunts the formation of the inflammasome-dependent cytokines IL-1β and IL-18 in response to stress, while inflammasome-independent cytokines are not affected (251). In addition, the depressogenic effect of LPS is attenuated in germ-free mice (256). Peripheral administration of the IL-6 receptor antibody MR16-1 counteracts the effect of psychosocial stress to disturb the gut microbiota and to evoke depression-like behavior along alterations in dendritic spine density and synaptic protein expression (257). Taken together with many other findings (252), it is thus emerging that gut microbiota and gut leakiness represent an important interface in the impact of stress on mood disorders.

## Impact of Intestinal Inflammation on Brain and Behavior

Ulcerative colitis and Crohn’s disease, the two major manifestations of IBD, are associated with an increased risk of mental disorders and can be exacerbated by stress. The disease impairs the quality of life not only in terms of GI symptoms, discomfort, and pain but also in terms of psychiatric comorbidities. Several mental disorders, including major depression, panic, and generalized anxiety, are more common in IBD patients than in community controls (258). Moreover, the psychiatric comorbidities can affect disease prognosis and response to treatment, as Crohns’ disease patients with major depression respond poorly to infliximab (259). In addition, stressful life events are risk factors for IBD development, exacerbation, and relapse (260). Some of the relationships between GI inflammation and emotional-affective disorders can be studied in experimental models of IBD in rodents. Colitis induced by dextran sulfate sodium (DSS) is immunologically similar to ulcerative colitis, while some immune processes occurring in trinitrobenzene sulfonic acid (TNBS)-induced colitis are reminiscent of those seen in Crohn’s disease (261). This range of chemically induced models of colonic inflammation is complemented by immunologically provoked paradigms of IBD. Thus, mice deficient in the antiinflammatory cytokine IL-10 (IL-10^−/−^) spontaneously develop a mild, patchy form of colitis which is highly dependent on the composition of the gut microbiota, given that the commensal *Akkermansia muciniphila* is able to induce colitis in IL-10^−/−^ mice (262).

### Cerebral and Behavioral Disturbances in Experimental Models of Inflammatory Bowel Disease (IBD)

Various experimental models of GI inflammation are associated with alterations in brain function and behavior (Table 2). While IL-10^−/−^ mice have been little studied in this respect, DSS-treated animals exhibit a number of behavioral alterations associated with intestinal inflammation, which have been linked to changes in gut–brain signaling (Table 2). For instance, mice with DSS-induced colitis are more anxious and less socially interactive than control mice, and these disturbances occur in parallel with increased circulating IL-6, IL-18, and NPY levels as well as with altered *Npy, Bdnf, Cox-2*, and *Mineralocorticoid receptor* gene expression in the brain, which points to an involvement of inflammatory and stress mechanisms in the behavioral perturbations (263). Moreover, DSS-treated mice display deficits in novel object recognition memory, which can be prevented by a probiotic (*Lactobacillus rhamnosus* R0011 and *Lactobacillus helveticus* R0052) and, thus, may involve a major influence of the GI microbiota on behavioral disturbances during colitis (264).

**Table 2:** Effects of gastrointestinal inflammation on emotional-affective and cognitive behavior.

Type of inflammation	Experimental design	Species (sex)	Behavioral effects	Additional effects	Reference
DSS-induced colitis	Three 7-day DSS cycles (3.5, 3, 3% w/v in drinking water) with 5-day recovery periods (tap water) in between	AKR mice (male)	Increased anxiety	DSS-induced anxiety prevented by vagotomy and by the probiotic *Bifidobacterium longum* NCC3001 given daily during and after DSS exposure for 14 d	(64)
DSS-induced colitis	11-day exposure to DSS (2% w/v) in drinking water	WT; NPY KO and PYY KO mice on mixed C57BL/6:129/SvJ (1:1) background (male and female)	Increased anxiety (male WT); increased depression-like behavior (female WT)	Decreased anxiety (female NPY KO and PYY KO); decreased depression-like behavior (male PYY KO)	(33)
DSS-induced colitis	7-day exposure to DSS (2% w/v) in drinking water	C57BL/6N mice (male)	Increased anxiety; decreased social interaction	Repeated WAS exposure (7 d) during DSS exposure prevents behavioral deficits	(263)
DSS-induced colitis	5-day exposure to DSS (5% w/v) in drinking water	Sprague-Dawley rats (male)	Increased anxiety; increased depression-like behavior	Resiniferatoxin-induced desensitization of colonic TRPV1 channels reverses behavioral deficits	(265)
DSS-induced colitis	5-day exposure to DSS (3% w/v) in drinking water	C57BL/6 mice (male and female)	Increased anxiety; decreased novel object recognition memory	Probiotics (mixture of *Lactobacillus rhamnosus* R0011 and *L. helveticus* R0052) administered daily for 7 days before and during DSS exposure) prevent behavioral deficits	(264)
TNBS-induced colitis	Single intrarectal administration of TNBS (10 mg in 50% ethanol)	NMRI mice (male)	Increased depression-like behavior	Nitric oxide synthase inhibition ameliorates depression-like behavior	(270)
*Trichuris muris* infection-induced colitis	Single infection with *Trichuris muris* (300 eggs/mouse)	AKR mice (male)	Increased anxiety-like behavior	Etanercept, budesonide and the probiotic *Bifidobacterium longum* NCC3001 normalize behavior	(275)

*DSS, dextran sulfate sodium; KO, knockout; NPY, neuropeptide Y; PYY, peptide YY; TNBS, trinitrobenzene sulfonic acid; TRPV1, transient receptor potential vanilloid-1; WAS, water avoidance stress; WT, wildtype*.

Besides enhanced anxiety, DSS-treated rodents also display aggravated depression-like behavior, a relationship that appears to be sex dependent (33, 265). The increase in anxiety- and depression-like behavior accompanying DSS-evoked colitis in rats is associated with increased firing rates of colonic afferents, which suggests that altered neuronal signaling is a major factor responsible for the colitis-induced behavioral changes (265). A similar conclusion can be drawn in mice in which DSS-evoked colitis is accompanied by increased anxiety-like behavior only if the vagus nerves are intact (64). In addition, DSS-evoked colitis alters the activity of central neurons. At the height of inflammation, stress-induced c-Fos expression, a marker of neuronal activation, is blunted throughout the corticolimbic system depending on housing conditions (266). Moreover, DSS-treated mice exhibit altered stress-associated behavior, increases in brain IL-6 and growth-regulated oncogene-α levels and brain region-specific changes in *corticotropin-releasing factor (Crf), Bdnf*, and *Npy* gene expression (267). In view of the emerging role of NPY in mediating stress resilience and treating post-traumatic stress disorder (268, 269) it appears worth investigating whether pharmacological manipulation of the NPY system has therapeutic effects in animal models of IBD which are sensitive to stress exposure.

Cerebral alterations associated with TNBS-induced colitis have been less thoroughly examined, but a relevant study has shown that TNBS-evoked colitis induces behavioral despair in mice (Table 2), which can be mitigated by inhibition of NO synthase (270). Potential effects of TNBS-induced colitis on other behavioral dimensions await to be explored, as TNBS-induced colitis has a number of cerebral effects such as increased excitability of hippocampal slices, altered hippocampal glutamatergic transmission and microglial activation, and lowered seizure threshold (271, 272). These effects appear to be cytokine and microglia dependent, because they are reversed by ICV administration of an anti-TNF-α antibody and minocycline, an inhibitor of microglial activation (273). In line with these findings, a positron emission tomography and *ex vivo* biodistribution study found increased levels of [^11^C]PBR28, an imaging biomarker of neuroinflammation, in the cerebellum 11 days after induction of colitis, suggesting activation of microglia or infiltration of macrophages in the brain (274).

Intestinal inflammation can also be triggered by parasites, and infection of mice with *Trichuris muris* has been found not only to cause mild to moderate colonic inflammation but also to elicit anxiety-like behavior (275). These effects are associated with increased circulating levels of TNF-α, IFN-γ, kynurenine and kynurenine/tryptophan ratio, and decreased hippocampal expression of *Bdnf* (275). Etanercept and, to a lesser degree, budesonide reduce cytokine and kynurenine levels and normalize behavior (Table 2) but do not influence *Bdnf* expression. By contrast, the probiotic *Bifidobacterium longum* NCC3001 normalizes both behavior and hippocampal *Bdnf* expression but does not affect circulating cytokine and kynurenine levels. Moreover, the anxiety-like behavior associated with *Trichuris muris* infection persists after vagotomy (275), unlike the anxiety-like behavior associated with DSS-induced colitis, which is prevented by vagotomy (64). Thus, the pathways and mechanisms underlying the behavioral perturbations in response to experimental colitis depend on the immunological triggers of GI inflammation.

### Intestinal Inflammation and Visceral Pain

IBD and experimental colitis are frequently linked to visceral and somatic hyperalgesia (276). Importantly, DSS-induced colitis enhances the spontaneous activity of spinal afferent neurons supplying the rat colon, and reduction of this spontaneous activity by intracolonic administration of lidocaine causes conditioned place preference in DSS-treated but not control rats (265). This finding indicates that colitis gives rise to continuous abdominal discomfort and not just to hyperalgesia toward acute stimuli (265). Continuous firing of afferent neurons from the inflamed gut is also likely to explain the increased expression of c-Fos and the enhanced phosphorylation of p42/44 MAPK in the lumbosacral spinal cord that takes place in mice suffering from DSS-induced colitis in the absence of noxious stimulation (277). Although inflammatory mediators are known to sensitize GI nociceptors, peripheral pain hypersensitivity alone does not explain all pain symptoms in IBD patients as some patients still suffer from pain in remission, and pain is poorly correlated with inflammatory markers (276, 278). Likewise, under conditions of experimental colitis in rodents, visceral hyperalgesia is not always going in parallel with inflammation. For example, DSS-induced colitis in mice leads to chemical and mechanical visceral hyperalgesia that persists for several weeks post-DSS treatment when intestinal inflammation has already subsided (279, 280). Similarly, mechanical visceral hyperalgesia associated with TNBS-induced colitis in rats can still be measured at a time when inflammation has resolved (281). Although a review of GI hyperalgesia mechanisms is beyond the scope of this article, it should not go unnoticed that TRPV1 ion channels play a role in post-inflammatory visceral hyperalgesia, since TRPV1 knockout and TRPV1 blockade prevent the development of mechanical visceral hyperalgesia after colitis (280, 282).

Chronic pain is a major internal stressor and as such a risk factor for the pathogenesis of anxiety and mood disorders (283, 284), a relationship that is also true for patients with Crohn’s disease in whom pain is associated with enhanced anxiety (285). TRPV1 desensitization in DSS-treated rats not only suppresses ongoing activity in spinal afferent neurons but also prevents the development of anxiety- and depression-like behavior associated with DSS-induced colitis (265). This observation confirms that nociceptive signaling from the colon is an important trigger for secondary changes in the brain that underlie the emotional-affective disturbances induced by experimental colitis.

### Gut Hormone Signaling in Intestinal Inflammation

Apart from immune and inflammatory mediators and nociceptive messages, gut hormones emerge as an important interface between GI inflammation, visceral pain, and perturbations of brain function, and behavior. IBD alters the expression of several GI and circulating gut hormones, including PYY and GLP-1 (39, 286–288), although the data are inconsistent due to heterogeneities in patient selection and assay methodology. Similarly, colonic PYY has been found to be reduced in response to TNBS in rats, while in response to DSS both increased and reduced colonic PYY levels have been reported (289–291). PYY can influence not only appetite but also emotional-affective behavior and visceral pain. Genetic deletion of PYY enhances depression-like behavior in mice, whereas anxiety-related behavior stays unaltered (33). Under conditions of DSS-induced colitis, PYY influences emotional-affective behavior in a sex-dependent manner, because anxiety-like behavior is modified only in female PYY knockout mice while depression-like behavior is altered only in male PYY knockout mice (33). Acute IP administration of PYY (3–36) induces schizophrenia-relevant behaviors including a profound impairment of social interaction (292). PYY is also involved in regulation of pain signaling, given that PYY knockout mice are more sensitive to somatic and visceral pain (293). In a wider perspective, these findings point to a potential involvement of PYY in the pain and emotional-affective alterations seen in animal models of colitis and IBD patients alike.

Like PYY, GLP-1 also influences emotional-affective behavior as revealed by the anxiogenic effect after acute administration and antidepressant effect after chronic administration of the GLP-1 analog, exendin-4, in rats (294). Other GLP-1 analogs have been reported to have an analgesic effect in both somatic and visceral pain states: ROSE-010 shows promising results in relieving visceral pain in patients suffering from IBS (295), while liraglutide suppresses the visceral hyperalgesia induced by LPS or water avoidance stress (WAS) in rats (296). In addition, intrathecally administered GLP-1 receptor agonists are able to alleviate pain evoked by peripheral nerve injury, bone cancer, and diabetes in rats and formalin-induced pain in rats and mice without affecting acute nociceptive responses (297). Therefore, PYY, GLP-1, and potentially other gut hormones emerge as targets for the control of GI inflammation, pain, and their relation to brain processes regulating emotion and affect.

### Gut Microbiota, Intestinal Inflammation, and Stress Responsivity

An emerging interface between GI immune system, GI inflammation and brain is posed by the gut microbiota and its multitude of signaling molecules. Germ-free mice display an exaggerated neuroendocrine response to restraint stress (298), and there is increasing evidence that the GI microbiota plays a role in the impact of stress on gut and brain (299). Mice lacking any microbiota display enlarged volumes of amygdala, hippocampus, and periaqueductal gray but a decreased volume of the anterior cingulate cortex compared to conventionally colonized mice (300, 301). These findings, together with marked alterations in brain neurochemistry, brain ultrastructure (300, 302, 303), brain microglia (26), and BBB function (28) may explain why germ-free mice display exaggerated stress responsivity (298), hyperactivity (302, 303), and visceral pain hypersensitivity (301). It would appear that the GI microbiota plays an important role in the development of the gut–brain axis, and that its disruption predisposes to maladaptive stress responsivity and behavioral profile (299, 304).

As discussed above, SCFAs seem to be important messengers of GI bacteria with a beneficial impact on the brain, and such a role may also be attributed to food, prebiotics, and probiotics that facilitate a physiological community structure of the GI microbiota. The prebiotic sialyllactose, for instance, has been shown to prevent the change in colonic microbiota composition, the increase in anxiety-like behavior and the decrease in hippocampal neurogenesis evoked by social disruption stress in mice (305). Fructooligosaccharides and galactosaccharides are likewise able to protect mice from the disruption of the microbiota community structure, the enhancement of anxiety- and depression-like behavior, and the rise of the circulating corticosterone concentration elicited by chronic psychosocial stress (306). The benefical effects of the prebiotics are associated with increased production of SCFAs in the gut (306).

Despite the clinical observations that stress has an adverse influence on IBD, *predictable* chronic WAS fails to modify the severity of DSS-evoked colitis in the mouse, but prevents colitis-evoked sickness behavior anxiety and disruption of social interaction (263). This effect of repeated WAS is associated with a rise of circulating corticosterone, an increase in hypothalamic *Npy* expression and a blockade of the colitis-associated induction of c-Fos in thalamus, hypothalamus, amygdala, and prefrontal cortex (263, 277). Likewise, the ability of colitis to amplify the expression of c-Fos in the lumbosacral spinal cord in response to noxious chemical stimulation of the colon is blunted by repeated WAS (277). In keeping with this finding, repeated WAS fails to aggravate mechanical and thermal hyperalgesia associated with DSS-induced colitis in mice (277) much as it fails to exacerbate visceral hyperalgesia associated with TNBS-induced colitis in rats (307). Taken together, these observations provide an explanation for the resilience effect of predictable chronic stress and show that the immune stress associated with experimental colitis alters not only brain function but also the cerebral processing of psychological stress and its impact on behavior. In addition, the influence of GI inflammation on stress processing in the brain is modified by environmental conditions, given that environmental enrichment appears to improve stress resilience as deduced from region-specific changes in the activity of the central amygdala, hippocampus, and infralimbic cortex (266).

## The Circle Completed: The Stressed Brain Facilitates Immune Activation and Inflammation in the Gut

### Adverse and Beneficial Effects of Stress on Intestinal Inflammation

Psychosocial stress is known to trigger disease exacerbation and relapses of IBD as well as IBS, a relationship that has been confirmed in animal models of GI inflammation (308). For instance, restraint stress amplifies the severity of colitis in IL-10^−/−^ mice as revealed by histopathology, increased expression of proinflammatory cytokines, and aggravated loss of body weight (309). Likewise, neonatal maternal separation stress enhances the permeability of the colonic mucosa both in wildtype and IL-10^−/−^ mice, but colitis develops only in IL-10^−/−^ mice (310), which attests to the multifactorial pathogenesis of chronic GI inflammation (311). DSS-treated mice exposed to chronic restraint stress (312), repeated psychological stress (313), repeated WAS (314), or a combination of repeated social defeat stress and overcrowding (315) also present with enhanced colonic inflammation scores, lowered body weight, and increased proinflammatory cytokine levels. Similarly, TNBS-induced colitis is aggravated after exposure to various stressors (316–319), which demonstrates an adverse impact of stress on disease course and severity. However, the outcome of the interaction between stress and GI inflammation is variable, depending on the type and duration of stress, the type of experimental inflammation and the experimental design. Repeated WAS, for example, has been shown to be without effect on acute and chronic DSS-induced colonic inflammation, on the one hand (263, 320), but to reactivate a quiescent chronic inflammation after exposure to DSS, on the other hand (314).

By contrast, there are also studies attesting to an ameliorating effect of stress on colonic inflammation. Cakir et al. (321) and Gülpinar et al. (322) both report that colitis severity is attenuated if animals are exposed to WAS during colitis induction, or if they are subjected to a controllable electric shock prior to colitis induction, respectively. In contrast to other experiments, these two studies used a single acute stressor, which raises the hypothesis that the acutely stressed brain and body are able to suppress inflammation. In fact, high concentrations of glucocorticoids released by the acute stress exposure are very likely responsible for the antiinflammatory action of stress, as deduced from the antagonistic effects of a glucocorticoid receptor antagonist (321, 322) and a CRF receptor antagonist (322). In line with this contention, chemical stimulation of the paraventricular hypothalamic nucleus, the major source of brain CRF, with glutamic acid alleviates TNBS-evoked colitis as measured by reduced colonic damage scores and blunted colonic levels of IL-6 and IL-17 (323).

In some animal studies, chronic stress *per se* has been shown to provoke the development of spontaneous colitis. In mice exposed to chronic subordinate colony housing, the colon displays an increased histological damage score, a higher number of inflammatory cells, and increased cytokine secretion (324, 325). Similar findings have been obtained in mice exposed to repeated social interaction stress, in which body weight loss, increased circulating cytokine levels and signs of colonic damage were observed (326). Changes in both glucocorticoid and cytokine signaling appear to be involved in the disease onset, but the relative importance of peripheral and central mechanisms remains to be investigated. It is not known whether chronic psychological stress may also trigger GI inflammation in humans. There are only some case reports that extensive physical stress such as that experienced by long-distance runners may give rise to reversible ischemic colitis (327, 328).

### Mechanisms Involved in the Impact of Stress on the Gut

**Figure 2:**
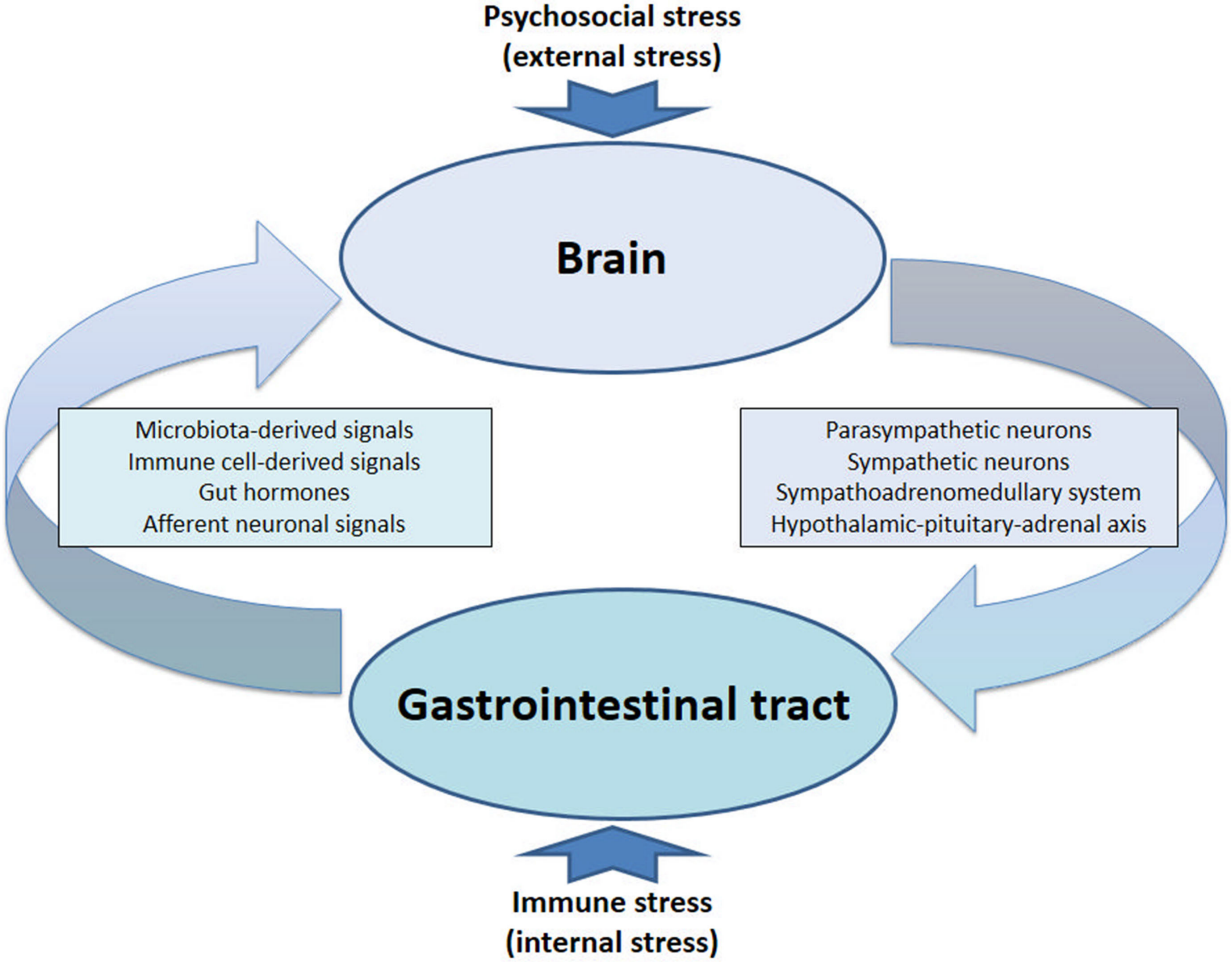
The circle completed: bidirectional communication between gut and brain under conditions of stress. The stressed brain facilitates immune activation and inflammation in the gut, while immune stress in the gut and periphery feeds back to the brain.

Psychosocial stress acts primarily on the brain and disturbs brain function in many ways (329), and these cerebral perturbations can adversely affect many peripheral organs, including the GI tract (Figure 2). Exposure to stress alters not only the activity of the HPA axis and its adrenocortical hormonal system but also that of the parasympathetic nervous system, the sympathetic nervous system, and the sympathetic adrenomedullary hormonal system (Figure 2). The HPA axis appears to be the most important stress response system (330) and represents a particularly important interface between stress and the gut, with CRF as a mediator operating both in the brain and GI tract (331). Glucocorticoids released from the adrenal cortex (cortisol in humans, corticosterone in rodents) dampen immune processes and are likely to interfere with immunological processes during stress. In addition, glucocorticoids can also induce non-neuronal catecholamine enzymes (332) which may add to the multiple signaling mechanisms of chronic stress exposure.

Both physical and psychological stressors cause formation of proinflammatory cytokines in the periphery (333), which may be due to “sterile inflammation” (334). In this process, damage-associated molecular pattern molecules, such as heat shock protein-72, uric acid, and ATP activate various PRRs (TLRs, NODs) that stimulate cytokine production (334). Importantly, there are individual differences in the sensitivity of the peripheral immune system that predict vulnerability to social stress (335). Specifically, IL-6 is most highly upregulated in mice that respond to chronic stress with exaggerated social avoidance behavior, whereas IL-6 knockout mice as well as mice treated with an IL-6 monoclonal antibody are resilient to social stress (335). These findings may have a bearing on stress-related psychiatric disorders as patients with treatment-resistant major depression display highly elevated serum levels of IL-6 (335).

In the gut, stress leads to disruption of mucosal tight junctions, which enhances mucosal permeability, facilitates microbial translocation, induces an immune response, and promotes inflammation (19). In addition, stress disrupts the community structure of the gut microbiota (253), which also weakens the mucosal barrier. The paracellular permeability through tight junctions of the GI mucosa is under the control of myosin light chain kinase (MLCK) which is involved in cytoskeletal regulation. MLCK can be activated by cytokines such as TNF-α, which enhances tight junction permeability by actomyosin contraction and reorganization of the tight junction (19). ML-7, a specific MLCK inhibitor, is able to blunt the increase of colonic paracellular permeability and the rise of LPS, ACTH, and corticosterone plasma levels evoked by partial restraint stress in rats (336).

CRF and NPY have proved to be important mediators of stress-related brain–gut interactions because both peptides occur at multiple sites in the gut and brain and affect various functions in both organ systems (32, 331, 337). CRF, for instance, participates in the stress-evoked inhibition of upper GI transit and stimulation of colonic motility (331). NPY serves a proinflammatory role in the gut, while cerebral NPY protects against distinct disturbances in response to immune challenge, enforcing stress resilience both in brain and periphery (337, 338). During restraint stress, fecal pellet output is significantly increased in mice deficient in NPY or the gut hormone PYY, relative to wildtype mice (338, 339). CRF_1_ receptor blockade reduces defecation in wildtype and NPY knockout mice but has no effect in PYY knockout mice (338). Endogenous NPY and PYY thus appear to inhibit the colonic motor stimulation evoked by stress, the effect of NPY depending on endogenous CRF acting via CRF_1_ receptors (338).

## Translational Implications

The information reviewed here reveals a bidirectional interplay between stress and the immune system, which is particularly obvious in the complex relationship between the GI immune system and the CNS. Psychosomatic medicine has long known that the digestive system is a preferred target of somatoform manifestations of stress. This is not, however, the end of the story. The stress-evoked disturbance of GI function, in which the cross-talk between the gut microbiota and the local immune system is of particular relevance, is signaled back to the CNS and may cause further disturbances of brain function. Thus, the impact of stress on the gut initiates a vicious cycle that is composed of both a brain–gut and gut–brain segment, the two segments being connected in the gut *via* the microbiota-immune network (Figure 2). This integrated view is important to consider in order to appreciate the mutual relationship between “stress and immunity” in a broad perspective.

The research we have reviewed here focusses on the impact of peripheral immune stress on the brain, given that a dysbalance of gut microbiota-immune homeostasis is thought to have a bearing on many neuropsychiatric disorders including Parkinson’s disease, multiple sclerosis, autism spectrum disorder, anxiety disorders, chronic fatigue syndrome, IBS, major depressive disorder, and cognitive decline (53, 55, 57, 82, 247, 250, 252, 299). Although research involving animal models provides compelling evidence for a causal relationship in an increasing number of instances, most evidence obtained from clinical studies is still of an associative nature. Thus, there is an appreciable gap in the translation of basic research to clinical applications across different microbe and host species. In addition, nutritional, environmental, genetic, epigenetic, and physiological factors will shape the microbiota-immune network in rodents in a quite different manner than that of humans. Furthermore, we still know little about the resilience of this GI network under changes of the external and internal environment, given that a disturbance of microbiota-immune homeostasis represents itself a local stress scenario.

The pathways along which peripheral immune stress is communicated to the brain are multifactorial, comprising both circulating molecules (microbe-derived molecules, immune mediators, gut hormones) and neuronal messengers. Through these signaling systems, several transmitter and neuropeptide systems within the brain are altered, enabling adaptive processes related to stress coping and resilience to take place or, if these measures are exhausted, giving rise to various CNS pathologies. Particular microbe-derived molecules (e.g., SCFAs), immune mediators (e.g., cytokines), and CNS messengers (e.g., neurotrophic factors, NPY) may play a particular role in determining whether inputs from the gut have a beneficial or deleterious effect on the brain. Dissection of the complex information flow from gut to brain will help identifying biomarkers of immune processes that carry a risk to stress the brain and unfold novel opportunities for therapeutic intervention.

## Author Contributions

PH conceived the article layout, wrote part of the review, integrated the parts written by the coauthors, and approved the final manuscript. AF, AMH, GZ, AJ, and FR assisted in the article layout, wrote part of the review, created the tables and figures, and approved the final manuscript.

## Conflict of Interest Statement

The authors declare that the research was conducted in the absence of any commercial or financial relationships that could be construed as a potential conflict of interest. The reviewer CJ and handling Editor declared their shared affiliation.
